# Prohibitins: A Critical Role in Mitochondrial Functions and Implication in Diseases

**DOI:** 10.3390/cells8010071

**Published:** 2019-01-18

**Authors:** Anna Signorile, Giuseppe Sgaramella, Francesco Bellomo, Domenico De Rasmo

**Affiliations:** 1 Department of Basic Medical Sciences, Neurosciences and Sense Organs, University of Bari “Aldo Moro”, 70124 Bari, Italy; 2 Water Research Institute (IRSA), National Research Council (CNR), Viale F. De Blasio, 5, 70132 Bari, Italy; giuseppe.sgaramella@ba.irsa.cnr.it; 3 Laboratory of Nephrology, Department of Rare Diseases, Bambino Gesù Children’s Hospital, Viale di S. Paolo, 15, 00149 Rome, Italy; francesco.bellomo@opbg.net; 4 Institute of Biomembrane, Bioenergetics and Molecular Biotechnology (IBIOM), National Research Council (CNR), 70126 Bari, Italy

**Keywords:** mitochondria, prohibitins, apoptosis, oxidative phosphorylation, mitochondrial dynamics

## Abstract

Prohibitin 1 (PHB1) and prohibitin 2 (PHB2) are proteins that are ubiquitously expressed, and are present in the nucleus, cytosol, and mitochondria. Depending on the cellular localization, PHB1 and PHB2 have distinctive functions, but more evidence suggests a critical role within mitochondria. In fact, PHB proteins are highly expressed in cells that heavily depend on mitochondrial function. In mitochondria, these two proteins assemble at the inner membrane to form a supra-macromolecular structure, which works as a scaffold for proteins and lipids regulating mitochondrial metabolism, including bioenergetics, biogenesis, and dynamics in order to determine the cell fate, death, or life. PHB alterations have been found in aging and cancer, as well as neurodegenerative, cardiac, and kidney diseases, in which significant mitochondrial impairments have been observed. The molecular mechanisms by which prohibitins regulate mitochondrial function and their role in pathology are reviewed and discussed herein.

## 1. Introduction

Mitochondria play a main role in both life and death of cells, ensuring a proper balance between pro- and anti-apoptotic factors and providing ATP through the oxidative phosphorylation (OXPHOS) system [1,2]. Therefore, mitochondria are sensitive to a variety of signals that are critical in regulating their functionality [3,4,5,6,7]. The regulation of mitochondrial function is an intricate process in which many resident and nonresident mitochondrial proteins participate. Prohibitin (PHB) proteins have attracted great attention in the last years due to their multiple functions in mitochondria. The first prohibitin (PHB1) was identified as an anti-proliferative protein in mammalian cells [8]. The second prohibitin (PHB2) was identified by its binding, with PHB1, to the IgM antigen receptor [9].

PHB1 and PHB2 have molecular weights of 32 and 34 kDa, respectively. Both are composed of an N-terminal transmembrane domain that is an evolutionarily conserved prohibitin domain common to other scaffold proteins (including stomatin, stomatin-like proteins 1, 2, 3, flotillin, and HflK/C) and a C-terminal coiled-coil domain involved in protein–protein interactions, including the interaction between themselves and with transcriptional regulators. PHB1 and 2 form a ring-like structure of approximately 1 MDa, consisting of about 12–20 PHB heterodimers, at the mitochondrial inner membrane. This complex has been identified in yeast [10,11,12], *Caenorhabditis elegans* (*C. elegans*) [13], and mammals [14].

Dysfunctions of PHB proteins have been associated with aging [13,15] and proliferative [16], degenerative [17,18], and metabolic diseases [19,20]. In mice, loss of the PHB complex results in embryonic lethality and, postnatally, in the degeneration of adult neurons [21] and loss of β cells [19]. At the cellular level, depletion of the PHB complex causes defects of proliferation and an increased sensitivity towards apoptosis [22,23,24]. PHB1 and PHB2 have been shown to localize in the nucleus, mitochondria, and cytosol, as well as associate with certain cell membrane receptors [25]. In the nucleus, PHBs, interacting with transcription factors, DNA-modifying associated enzymes, cell cycle associated proteins, and RNA-binding proteins, have a role as transcriptional co-regulators [26]. In the cytosol, PHBs interact with proteins involved in cytoskeletal transport and cellular signaling, and also with cell membrane proteins and receptors [26]. However, it is not known whether PHBs heterodimerize in these cellular compartments. Evidences suggest that PHB1 and PHB2 function primarily within mitochondria [23,27]. In fact, PHB proteins are highly expressed in cells with a high energy demand, which present more susceptibility towards mitochondrial dysfunction. It has been shown that human PHB2 contains an uncleavable mitochondrial targeting at the N-terminus and a signal for nuclear localization at the C-terminus [23]. In contrast, in human PHB1, the N-terminus, even if it is necessary for mitochondrial localization, does not possess a typical mitochondrial targeting sequence [23]. The Akt-dependent phosphorylation of PHB1 on Thr258 promotes its mitochondrial translocation [28].

PHBs have been found to be implicated in mitochondrial respiratory chain subunit degradation, assembly and activity of the oxidative phosphorylation system (OXPHOS), mitochondrial biogenesis, mitochondrial networks, mitochondrial apoptosis, and mitophagy.

Due to their multiple functions in mitochondria, PHBs have been found to be altered in various pathological conditions, making them a potential target for therapeutic approaches.

## 2. Role of Prohibitins in the Oxidative Phosphorylation System

The OXPHOS system consists of the respiratory chain complexes NADH-ubiquinone oxidoreductase (complex I), succinate-ubiquinone oxidoreductase (complex II), ubiquinone-cytochrome *c* oxidoreductase (complex III) and cytochrome *c* oxidase (complex IV), plus the F_1_F_o_-ATP synthase (complex V) [1]. Protein components of the OXPHOS system are encoded in a coordinated expression by the two cellular genomes (nuclear DNA and mitochondrial DNA). Mitochondrial DNA encodes for 13 proteins of the OXPHOS system, while hundreds of proteins involved in respiration and in different mitochondrial functions are encoded by nuclear DNA [1]. The structure and function of the OXPHOS system depend on the correct synthesis, transport, and assembly of the mitochondrial proteins encoded by both nuclear and mitochondrial genomes [1].

**Figure 1:**
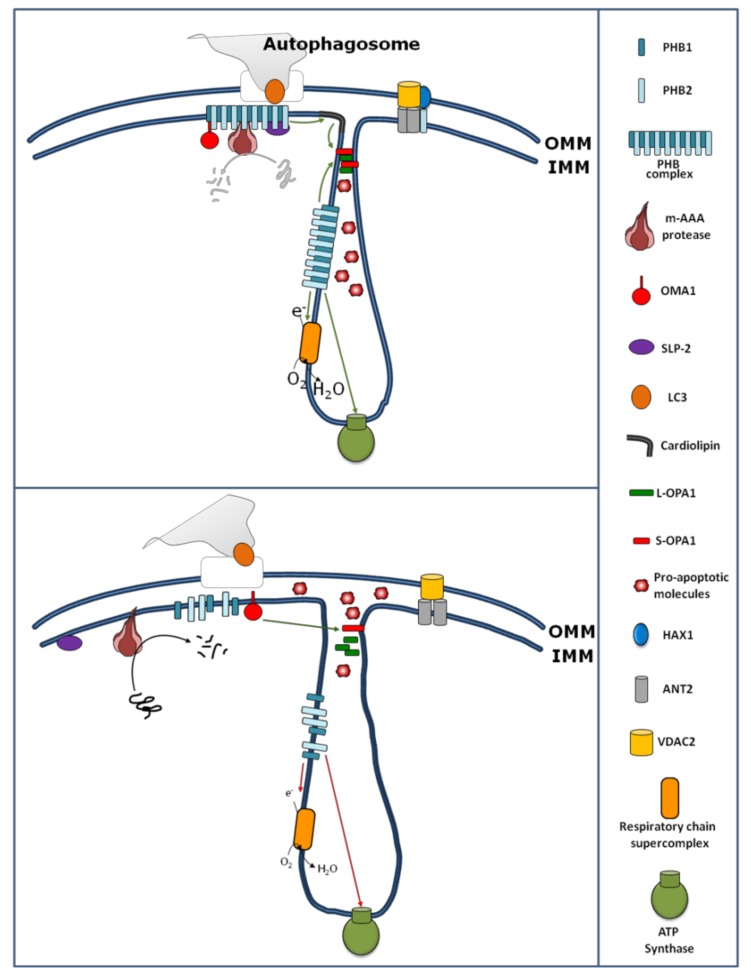
Role of the PHB complex and PHB proteins in mitochondrial functions. (**Upper panel**) The PHB complex interacts with *m*-AAA [11] and OMA1 proteases [30], inhibiting their activity, and with SLP-2 protein [31], resulting in a reciprocal protein stabilization. The stabilization of the PHB complex affects the maturation of cardiolipin [32] that, in turn, together with the PHB complex, promotes the stabilization of OPA1 [24,33] and the formation of the respiratory chain supercomplex [34,35]. The PHB complex also ensures a correct biogenesis of the ATP synthase [36]. PHB2 protein also has a role as a receptor for selective autophagy (mitophagy) by interacting with LC3 through its LC3-interacting domain [37]. Independently to its binding to PHB1, PHB2 forms another complex with HAX1, VDAC, and ANT3 [23]. (**Lower panel**) The down-regulation of PHBs and the loss of the PHB complex results in the activation of *m*-AAA [11] and OMA1 [30] proteases, degradation of SLP-2 [31], and a defect in cardiolipin maturation [32]. The increased *m*-AAA protease activity alters the turnover of unassembled mitochondrial respiratory chain subunits [11]. The increased activity of OMA1 [30] and the altered maturation of cardiolipin [32] result in an increased processing or decreased stability of L-OPA1 [33]. The altered balance between L-OPA1 and S-OPA1 causes mitochondrial fragmentation and alteration of mitochondrial cristae ultrastructure, with a consequent release of pro-apoptotic molecules from the intermembrane space, leading to apoptosis [38]. As with OPA1, the down-regulation of PHB2 leads to proteolysis of HAX1, which is implicated in the mitochondrial permeability transition during apoptosis [23]. OMM: Outer mitochondrial membrane; IMM: Inner mitochondrial membrane.

The PHBs are involved in the regulation of OXPHOS activity by physical interaction with some subunits of the OXPHOS complexes, as well as by modulating their stability and translation. Indeed, one of the first functions attributed to PHBs within mitochondria is their role in the mitochondrial protein quality control. Instability of mitochondrial-encoded subunits of the respiratory chain has been observed in the absence of PHB1 [11,14,29]. In yeast, over-expression of the PHB complex results in the stabilization of newly synthesized subunits encoded by the mitochondrial DNA, suggesting that this complex might function as a holdase/unfoldase chaperone which binds the mitochondrial translation products and protects them from proteolysis [14]. Moreover, the PHB complex regulates the proteolysis of unassembled inner membrane proteins of the OXPHOS system by forming a supercomplex with *m*-AAA protease (Figure 1).

The proteolysis is accelerated in phb-null yeast cells [11], suggesting that the PHB-*m*-AAA supercomplex results in inhibition of *m*-AAA activity. More recently, in yeast, an interaction between the PHB complex and Pim1, a Lon-like protease, has been observed showing a regulatory effect on the stabilization of the unassembled Atp7 subunit of the F_1_F_o_-ATP synthase during the biogenesis of this complex [29].

In mammals, PHB1 down-regulation by siRNA transfection results in the reduced expression levels of some complex IV subunits encoded by both mitochondrial and nuclear DNAs, associated with a decrease of the enzymatic activity [39]. Other studies report that silencing of PHB1 or PHB2 causes a reduced activity of the other respiratory chain complexes [19,21,40,41,42], and furthermore, the over-expression of PHB1 increases complex I-dependent mitochondrial respiration [43] and promotes ATP formation [43,44]. Prohibitins have also been shown to physically interact with subunits of complex IV in yeast [14] and with subunits of complex I in mammals [45]. In agreement with these results, other findings support the role of prohibitins in the stability of OXPHOS subunits as shown in the HeLa cell line, where the depletion of stomatin-like protein 2 (SLP-2), a protein of the mitochondrial inner membrane that interacts with PHBs, induces an increase in the proteolysis of PHBs and some subunits of complexes I and IV [31]. 

Beyond the role of PHBs in the stability/proteolysis of OXPHOS system subunits, PHBs also contribute to mitochondrial protein translation [46]. In mouse embryonic fibroblast (MEF) cells, reduced expression of PHB1 or PHB2 by dsRNA or PHB2 gene excision, respectively, causes a reduction of mitochondrial protein synthesis [46] according to the findings, showing that the mitochondrial translational machinery is closely associated with the mitochondrial inner membrane [47]. In this view, it has been recently shown that SLP-2, beyond the interaction and stabilization of PHBs (Figure 1), also interacts with mitoribosomes, regulating, in this way, the mitochondrial translation [48].

PHB2 binds with high affinity to the sphingosine-1-phopshate that is produced in the mitochondria by sphingosine kinase 2 (SphK2). It has been shown that SphK2-null mice present aberrant COX enzymes due to altered interaction between subunit IV of complex IV and PHB2, leading to mitochondrial dysfunction [49].

PHB1 also interacts with the signal transducer and activator of transcription 3 (STAT3). This is a transcription factor that controls cell growth and responses to inflammation and cellular stress. Additionally, a pool of STAT3 resides in the mitochondria [50], where it regulates mitochondrial respiration through the interaction with complexes I and II [50,51]. The interaction in mitochondria between STAT3 and PHB1 prevents, in epithelial cells, the mitochondrial dysfunction in inflammatory bowel diseases [52].

In addition to the role of PHBs on the stability/proteolysis and translation of subunits of the OXPHOS system, some data suggest a role for PHBs in the assembly of OXPHOS system complexes [36] and in formation of a supercomplex [35,42] (Figure 1). The assembly of F_1_F_o_-ATP synthase appears to be particularly sensitive to the loss of prohibitins [36].

Thus, PHBs have an impact on the OXPHOS system (i) by protecting the newly synthesized subunits from proteolysis, as a chaperone and/or by inhibitory effects on *m*-AAA protease, (ii) by physical interaction with some subunits of the OXPHOS system, and (iii) by promoting mitochondrial protein translation and supercomplex assembly.

## 3. Role of Prohibitins in Mitochondrial Biogenesis

Mitochondrial biogenesis is the process by which cells increase their individual mitochondrial mass by growth and division of pre-existing mitochondria. Peroxisome proliferator-activated receptor-gamma coactivator-1alpha (PGC-1α) is a crucial co-transcriptional regulation factor that induces mitochondrial biogenesis by activating other transcription factors, such as nuclear respiratory factors 1 and 2 (NRF1 and NRF2), which in turn control the expression of many nuclear genes encoding for structural proteins of the OXPHOS system, mitochondrial import complexes, enzymes of heme biosynthesis, and mitochondrial transcription factor A (TFAM) [53].

The brown and white adipose cell differentiation is a typical event in which an increase in mitochondrial biogenesis occurs. In 3T3-L1 preadipocytes, the differentiation has been associated with increased levels of PHB1 and PHB2 proteins. In contrast, knockdown of PHB1 or PHB2 results in reduced expression of adipogenic markers, accumulation of lipids, and reduction of mitochondrial content [54]. Moreover, transgenic female mouse models over-expressing PHB1 in adipocytes show development of obesity associated with up-regulation of mitochondrial biogenesis [55]. In this study, it is reported that PHB1 over-expression results in increases of mitochondrial DNA copy number and several resident and nonresident mitochondrial proteins, such as PGC-1α, subunit A of succinate dehydrogenase, subunit 1 of complex IV, DNA polymerase subunit gamma, NRF2, optic atrophy 1 protein (OPA1), dynamin-related protein (DRP1), and TFAM [55]. Due also to the nuclear localization of PHBs, where they interact with several transcription factors [56], the authors suggested an increased nuclear–mitochondrial communication in adipocytes over-expressing PHB1 [55]. However, the molecular mechanism by which PHBs can induce an increase of mitochondrial biogenesis still needs further investigation. Beyond its role as the expression inducer of PGC-1α, data suggest that PHB1 regulates the copy number of mitochondrial DNA by stabilizing TFAM proteins in a chaperone-like manner [57]. In HeLa cells, in fact, RNAi-mediated down-regulation of PHB1 or PHB2 results in an altered organization and reduced mitochondrial DNA copy number, and predisposes cells to apoptosis [57]. In a brief summary, PHBs can induce mitochondrial biogenesis by promoting the expression of proteins encoded by both nuclear and mitochondrial DNA, via PGC-1α and TFAM, respectively.

## 4. Role of Prohibitins in Unfolded Protein Response (UPR^mt^)

Mitochondrial proteins, encoded by both the mitochondrial and nuclear genomes, are folded and continuously subjected to a quality control process by heat shock proteins, chaperones, and proteases [58] to maintain correct mitochondrial proteostasis. Under stress conditions, such as depletion of mitochondrial DNA, loss of mitochondrial membrane potential, imbalance between nuclear- and mitochondrial-encoded proteins, or accumulation of unfolded proteins within the mitochondria, an activation of a retrograde signaling process, known as the unfolded protein response of the mitochondria (UPR^mt^) occurs. The classical UPR^mt^ consists of a re-localization of some proteins from the mitochondria to the nucleus, resulting in a transcriptional reprogramming regarding the expression of mitochondrial chaperones, proteases, and antioxidant enzymes in order to restore mitochondrial function (reviewed in [59]). Interestingly, an increased level of the PHB complex has been reported under UPR^mt^ activation as depending on imbalance in the synthesis of mitochondrial- and nuclear-encoded mitochondrial proteins, in mammals [60] and *C. elegans* [13], or on accumulation of unassembled subunits of respiratory chain, in yeast [61]. In *C. elegans*, it is also reported that the lack of PHB induces a strong UPR^mt^ activation (reviewed in [62]). This enforces the role of PHBs in the mitochondrial protein quality control.

## 5. Role of Prohibitins in Mitochondrial Dynamics and Ultrastructure

Mitochondria are highly dynamic structures that fuse (fusion) and divide (fission) continuously, adjusting their shape and cellular distribution depending on cell type and the energy demands of the cell. Several proteins, localized in the outer and inner mitochondrial membranes, are involved in the fusion and fission events, including mitofusins 1 and 2 (Mfn1, Mfn2), and OPA1, required for mitochondrial fusion, and DRP1 and fission 1 (Fis1), required for mitochondrial fission [63].

Loss of prohibitins has been shown to affect mitochondrial morphology in *C. elegans*, where prohibitin depletion resulted in fragmented and disorganized mitochondria [13]. Similarly, loss of prohibitins in mouse embryonic fibroblasts (MEFs) resulted in an increased mitochondrial fission and the loss of mitochondrial cristae [24]. Recently, in cardiomyocytes, a study showed that miR-361 initiates mitochondrial fission, with consequent apoptosis leading to myocardial infarction, through the suppression of PHB1 translation [64]. A possible mechanism for PHB-dependent mitochondrial fragmentation comes from the discovery that the PHB complex, at the inner membrane, is required for OPA1 stability (Figure 1) [24]. OPA1 is involved in mitochondrial fusion, remodeling of cristae structure, and apoptosis. OPA1 undergoes constitutive processing, leading to the conversion of an uncleaved long OPA1 (L-OPA1) into a cleaved short OPA1 (S-OPA1) form [51]. The balance between the L and S forms contributes to determining the cell fate [63,64,65]. Different mitochondrial proteases, such as *m*-AAA, OMA1, and YMEL1 cleave OPA1 from its L to S form [66,67]. An increase of cleavage of OPA1, from the L-OPA1 to S-OPA1 forms, has been found after disruption of interactions between *m*-AAA and PHB1 [68]. In this work, Sato and colleagues [68] treated HeLa cells with aurilide, a marine natural product derived from *Dolabella auricularia*. The aurilide, by binding to PHB1, caused the PHB-*m*-AAA complex dissociation associated with OPA1 processing, mitochondrial fragmentation, and apoptosis similarly to the cells silenced for PHBs. Additionally, the stable HeLa cell line expressing a mutant form of OPA1 resistant to proteolysis was protected from aurilide-induced apoptosis, indicating that the PHB1-*m*-AAA complex contributes to apoptosis via OPA1 processing [68]. In this work, the PHB-dependent protease responsible for OPA1 processing has not been identified, even if *m*-AAA protease might be a good candidate. This comes from the finding showing that the PHB complex inhibits *m*-AAA protease by binding it [11]. However, other proteases cannot be ruled out. In fact, depletion of both PHB1 and PHB2 could stabilize an active form of OMA1 that, in turn, processes OPA1 [11,69] (Figure 1).

Additionally, mitochondrial shape and ultra-structure can also be affected by the lipid composition of mitochondria. PHBs affect the maturation of cardiolipin (Figure 1) [32]. Cardiolipin is a phospholipid of the mitochondrial inner membrane that plays a role in the OPA1-dependent mitochondrial fusion [33]. It has been proposed that after the mitochondrial outer membrane fusion (mediated by mitofusin), cardiolipin and OPA1 work together to fuse the mitochondrial inner membranes [33]. Furthermore, in yeast, data from proteomic approaches reveal that Mdm33, a protein involved in the regulation of mitochondrial structure, dynamics, and homeostasis of mitochondrial phospholipids like phosphatidylethanolamine and cardiolipin, interacts with PHB1 and PHB2 [70]. 

The role of PHBs in the stability of OPA1, likely as a chaperone, and in cardiolipin maturation and distribution, likely as a scaffold (see below), contributes to the mitochondrial fusion and restructuration of mitochondrial cristae.

## 6. Role of Prohibitins in Apoptosis

During apoptosis, many cellular events occur. A key step for initiating mitochondrial apoptosis is the release of cytochrome *c* from mitochondria to the cytosol, associated with the restructuration of mitochondrial cristae. Over-expression of PHB1 has been found to protect cardiomyocytes from hypoxia-induced cell death by inhibiting cytochrome *c* release and decreasing levels of Bcl2 protein [71]. In addition, over-expression of PHB1 in cardiomyocytes has been shown to protect cells from oxidative stress-induced mitochondrial apoptosis, and preserves the cytochrome *c* release and mitochondrial membrane permeability [44]. Vice versa, in *Phb2*^−/−^ MEFs, the absence of the PHB complex increases the sensitivity towards apoptotic stimuli by promoting the cytochrome *c* release [24].

Cytochrome *c* release, and thus mitochondria-dependent apoptosis, is a process that is also influenced by the mitochondrial membrane phospholipid composition that, in turn, might depend on the scaffolding function of the PHB complex [32,72]. Cardiolipin plays an important role in the cytochrome *c* release, and also provides an anchor and platform for caspase-8 activation at the mitochondrial surface [73]. In mammalian cells, it has been observed that prohibitins, interacting with DNAJC19 protein, a subunit of the translocator of the mitochondrial inner membrane required for ATP-dependent import of mitochondrial pre-proteins, regulate cardiolipin remodeling. It has been proposed that the PHB/DNAJC19 complex facilitates tafazzin-dependent acylation of monolysocardiolipin, generating cardiolipin and ensuring a correct lipid distribution in mitochondrial membranes [32]. In agreement, DNAJC19-deficient mitochondria show alterations in the acyl chain compositions of mitochondrial membrane lipids, affecting cristae morphology [32].Similarly, in MEF cells, depletion of PHB2 reveals severe defects in cristae formation [24]. Cardiolipin also has a role in the formation and/or stability of the prohibitin-*m*-AAA protease complex. In yeast, deletion mutants of the *tafazzin* gene had decreased cardiolipin content, associated with a reduced content of the prohibitin-*m*-AAA protease complex [74]. Furthermore, cardiolipin and PHBs are involved in biogenesis and stability of the OXPHOS system and supercomplex formation [34,75].

The restructuration of the mitochondrial cristae is an important aspect that occurs during the early stages of apoptosis, which involves OPA1 and the F_1_F_o_-ATP synthase complex. OPA1, independent of its role in mitochondrial fusion, also undergoes oligomerization along the cristae junctions [38]. During apoptosis, OPA1 oligomers are destabilized, with consequent cristae opening and cytochrome *c* exit from mitochondria [38,76]. This mechanism appears to be dependent on F_1_F_o_-ATP synthase dimerization [77]. It should be recalled that OPA1 and the F_1_F_o_-ATP synthase complex are particularly sensitive to PHB depletion [24,29,36]. Interestingly for the structure of mitochondrial cristae, the analysis of the purified yeast PHB complex by single-particle electron microscopy reveals a ring-like structure with an outer diameter of about 200–250 Å [12]. It has been speculated that in addition to the PHB complex integrated in the inner mitochondrial membrane, due to the similar diameters of the PHB complex and cristae tubules, the PHB complex might be assembled perpendicular to the axis of the cristae, contributing to cristae stabilization and assuring a barrier against the diffusion within cristae membranes [72]. 

PHB2 also modulates the stability of the anti-apoptotic HCLS1-associated protein X-1 (HAX1) protein. Reduced expression of PHB2 has been found to cause a decrease in mitochondrial HAX1, associated with a loss of mitochondrial integrity and the activation of caspase 9/caspase 3 and apoptosis [23]. The down-regulation of PHB2, like for OPA1, leads to proteolysis of HAX1 [23]. Independently of its binding to PHB1, PHB2 appears to form another complex with HAX1, VDAC and ANT3 (Figure 1) [23]. VDAC and ANT3 are implicated in the mitochondrial permeability transition during apoptosis.

The role of PHBs in apoptosis also extends to their regulation at the translational level. miRNA-23A has been found to be upregulated during oxidative stress [78]. This miRNA increases the transcriptional activity of p53 that, in turn, promotes the expression of another miRNA, miRNA-128. This suppresses the expression of PHBs, promoting apoptosis [78]. At the same time, studies have shown that PHB1 is present in the nucleus of several breast cancer cell lines, where it can bind to p53 and stimulate its transcription [78]. PHB1 is also capable of modulating the expression of genes encoding for proteins involved in apoptosis [56,79,80] and co-localizing in the nucleus with many transcription factors [81] that influence the cell cycle and growth. The anti-apoptotic role of PHBs in mitochondria is underlined by the high level of the PHB complex within mitochondria in cancer cells [16]. Furthermore, PHB1 accumulates in mitochondria of melanoma cells after anticancer treatment, leading to chemoresistance [82], and an elevated PHB1 level is reported in activated lymphocytes, characterized by resistance towards apoptosis, of patients affected by multiple sclerosis [83].

This scenario supports the anti-apoptotic role of PHBs in mitochondria, as well as the increased susceptibility towards apoptosis in the absence of PHBs.

## 7. Prohibitins in Mitophagy

Autophagy is a process of self-eating, whereby cytosolic constituents are enclosed by a double-membrane vesicle (autophagosome) before delivery to the lysosome for degradation [84]. This takes place in several cellular processes, like development, cellular homeostasis, tumor suppression, and aging. Microtubule-associated protein 1 light chain 3 beta (LC3) associates with the autophagosome, participating in the final stages of its formation [84]. Mitophagy represents a process by which damaged mitochondria are specifically removed from the cell [84]. It is generally accepted that the recognition of damaged mitochondria, by LC3, depends on outer mitochondrial membrane receptor proteins [84]. Recently, a role of PHB as a receptor for selective autophagy (mitophagy) has been hypothesized [37]. It has been found by Levine and collaborators that PHB2, at the inner mitochondrial membrane, is involved in recognition by LC3 [37]. In particular, it has been shown that upon rupture of the outer mitochondrial membrane, through a proteasome-dependent mechanism, PHB2 becomes available for interaction with LC3 through its LC3-interacting domain (Figure 1) [37]. This adds a new role for PHB2 in mitochondria biology.

## 8. Pathological Role of Prohibitins

The data described underline the critical roles played by PHB proteins in multiple aspects of mitochondrial biology. PHB deregulation has been associated with physiopathological conditions such as aging [15], neurodegenerative diseases [17,18], kidney diseases [20], cardiac diseases [64], and cancer [16].

### 8.1. Prohibitin and Aging

The aging process is a well-studied field in which one universal point of view is the involvement of mitochondrial decline in terms of activity, biogenesis, structure, morphology, and increase of ROS production. In this regard, studies display that prohibitin-deficient yeast cells show a reduced replicative lifespan [85], associated with mitochondrial decline and characteristic morphological changes of aging [85].

The nematode *C. elegans* is extensively used as a model of the aging process [86]. In *C. elegans*, PHB1 and PHB2 depletion by RNAi results in delays in development and reductions in body size associated with a wide range of somatic and germline defects [13,15]. Interestingly, the mitochondrial PHB complex has been shown to modulate longevity depending on the metabolic status of the worms [13,15]. Deletions of PHB1 or PHB2 shorten the lifespan of nematodes, as shown in yeast, while they extend the lifespan of a variety of *C. elegans* mutants [13,15], such as mutants of insulin/insulin growth factor 1 or transforming growth factor beta signaling, mutants with altered fat metabolism, mutants with defective mitochondrial electron transport chains, and dietary-restricted animals. It has been shown that in wild type animals, PHB depletion, causing mitochondrial defects, activates a retrograde cellular response, leading to an increase in fat content and an over-proliferation of malfunctioning mitochondria that, in turn, increases reactive oxygen species production, exacerbating metabolic defects and cellular damage, and reducing lifespan. In mutated worms, where the proliferation of mitochondria is inhibited, the absence of PHBs switches the cellular metabolism to fat utilization, leading to longevity [13,15].

In mammals, recent metabolic labeling studies showed that matrix arm subunits of complex I of the mitochondrial respiratory chain had shorter half-lives than membrane arm proteins in the liver and heart [87], and in HEK cell lines [88]. This is in agreement with the hypothesis that matrix arm proteins might exist as free monomers [89] or in less stable, smaller complexes [87], and with the finding showing that newly imported subunits of the matrix arm can be assembled in complex I in exchange with the “older aged” ones already assembled to the complex [90,91]. Data of the abundance of complex I matrix subunits have been correlated with ROS production [92]. In particular, the data show that young longer-living mice presented low levels of matrix arm subunits of complex I when compared to old or shorter-living mice, while the levels of membrane arm subunits remained unchanged [92]. The authors suggest that the low levels of matrix arm subunits did not allow for the assembly of a further smaller sub-complex able to oxidize respiratory substrates without the coupler between electron transport and proton pumping, thus producing ROS and making the respiratory chain less efficient. The levels of matrix arm subunits appear to be associated with the protective effects of chaperone-like functions of PHBs, because PHB1 levels reflected the changes observed for matrix arm subunits [92]. Therefore, the increased amounts of complex I matrix arm subunits in old and shorter-living mice or the decreased amount in young-living mice might be due to a decreased or increased proteolytic degradation, respectively [92]. This suggests a possible long-term dangerous effect of long-term PHB-mediated stabilization of matrix arm subunits of complex I. These subunits, in excess, could assemble to form a sub-complex that, producing superoxide on their own and diminishing the efficiency of substrate utilization, can contribute to age-dependent mitochondrial decline. Interestingly, in human cells silenced for the complex I assembly factor NDUFAF1, harboring a 40% decrease of fully assembled complex I [92,93] and a 50% decrease of its activity [94], the PHB1 knockdown rescues complex I assembly and activity [92], reducing the complex I-dependent ROS production [92]. Probably, in cells with compromised complex I assembly, a subcomplex-generating ROS of complex I persists, and the knockdown of PHB1 allows for the proteolytic degradation of the subunits of this subcomplex-generating ROS, not only reducing the ROS production but also allowing a little increase in the efficiency of substrate utilization. This is in agreement with the findings in *C. elegans*, in which PHB depletion increases lifespan in mutants with compromised respiratory chains [15]. This is an intriguing aspect, considering that several human pathological mutations in genes encoding for structural peripheral subunits of complex I lead to the formation of a 200 kDa sub-complex able to oxidize NADH without proton pumping (for review, see [94]). Interestingly, in KO-NDUFS4 mouse models harboring complex I deficiency and presenting a 200 kDa subcomplex able to oxidize NADH [95], the treatment with rapamycin, which is reported to decrease the PHB1 protein level [92], ameliorated the phenotype [95].

Taken together, these results suggest a role of PHBs as having holdase/unfoldase chaperone activity in the aging process.

Notably, the age-dependent decline of mitochondrial activity in rat skeletal muscle [96] and the increased mitochondrial biogenesis in the development of obesity in PHB over-expressing mice [55] have been found to be more pronounced in females than in males. It should be noted that PHBs also interfere with sexual hormone receptors [23].

### 8.2. Prohibitins in Parkinson’s and Alzheimer’s Diseases

PHBs interact with OPA1 and *m*-AAA proteases in order to maintain an exact cristae morphology and regulate the mitochondrial protein expression levels. Mutations in the *OPA1* gene cause neurodegeneration in autosomal dominant optic atrophy [97], while mutations in *m*-AAA protease subunits cause spinocerebellar ataxia, hereditary spastic paraplegia, and a spastic-ataxia neuropathy syndrome [98]. It goes without saying that PHB loss can be deleterious for neuron cells. In fact, on the contrary to tumor cells, where PHB over-expression appears to give resistance to apoptosis, decreased PHB levels have been found to be involved in the pathogenesis of Parkinson’s (PD) and Alzheimer’s diseases (AD) where they give more susceptibility to apoptosis.

*Parkinson disease*: In sporadic PD, the most common human neurodegenerative movement disorder, decreased activity of complex I and oxidative damage to subunits of complex I have been found in dopaminergic neurons of patients’ autoptic samples of substantia nigra [99], and in fibroblast cell cultures from patients [100] of sporadic and familial PD. Another work reports a reduction of PHB and a subunit of ATP synthase in the substantia nigra of five PD post mortem human brains [17]. It is worth noting that PHBs have been found to modulate the expression of the ATP synthase complex [36]. These findings were confirmed by Dutta et al. [18] and, in addition, PHB reduction has been found in MPP^+^-induced cellular models of PD, associated with mitochondrial dysfunction. In this model, the over-expression of PHB protected cells from MPP^+^-induced neuronal death by preventing decreases in mitochondrial membrane potential, ROS production, and release of cytochrome *c* from mitochondria to the cytosol [18]. Considering also the altered mitochondrial structure found in PD [101], a role of PHBs in the maintenance of mitochondrial cristae structure and susceptibility towards apoptosis might be speculated in PD.

*Alzheimer’s diseases*: The impairment of OXPHOS has been extensively documented in cell lines, animal models, and postmortem brains, revealing a main role of mitochondrial dysfunction in AD synapse failure [102]. Up till now, research has failed to find changes in the expression levels of prohibitin in the frontal cortex in both early and later stages of sporadic AD [17]. However, a disruption of the PHB complex has been observed during Alzheimer’s disease progression in the olfactory bulb [103]. Furthermore, conditional PHB2 gene ablation in mice showed a post-natal massive neurodegeneration, associated with aberrant mitochondria accumulation and hyperphosphorylation of the microtubule-associated protein tau [21]. Beyond the decrease of respiratory chain activity, studies showed the accumulation of autophagosomes and other pre-lysosomal autophagic vacuoles in AD patients’ brains [104], suggesting the possibility to explore the role of PHB2 in mitophagy in AD. 

*Alzheimer’s diseases*: The impairment of OXPHOS has been extensively documented in cell lines, animal models, and postmortem brains, revealing a main role of mitochondrial dysfunction in AD synapse failure [102]. Up till now, research has failed to find changes in the expression levels of prohibitin in the frontal cortex in both early and later stages of sporadic AD [17]. However, a disruption of the PHB complex has been observed during Alzheimer’s disease progression in the olfactory bulb [103]. Furthermore, conditional PHB2 gene ablation in mice showed a post-natal massive neurodegeneration, associated with aberrant mitochondria accumulation and hyperphosphorylation of the microtubule-associated protein tau [21]. Beyond the decrease of respiratory chain activity, studies showed the accumulation of autophagosomes and other pre-lysosomal autophagic vacuoles in AD patients’ brains [104], suggesting the possibility to explore the role of PHB2 in mitophagy in AD. 

Evidence has been provided showing that prohibitins are substrates for transglutaminase 2 [105], a multi-functional enzyme which is implicated in the pathogenesis of several diseases including Parkinson’s disease and Huntington’s disease. This suggests that prohibitin could be considered a potential therapeutic target for these neurological diseases. 

### 8.3. Prohibitins in Kidney Diseases

Mitochondria are increasingly recognized as key players in genetic and acquired renal diseases, and renal disease has been reported in patients with genetic defects involving mitochondrial respiratory chain assembly factors, CoQ10 biosynthesis, mtDNA translation, and mtDNA maintenance [106]. Podocytes, which are essential components of the kidney filtration unit and responsible for ultrafiltration of protein-free urine from plasma, have the most abundant mitochondrial network among glomerular cells [107]. In a podocyte-specific PHB2 knockout mouse model, proteinuria, glomerulosclerosis, end-stage renal failure, and animal death within 4–5 weeks have been observed. In agreement with the role of PHB2 in mitochondria, after 3 weeks, the animals presented disorganized lamellar cristae structures [20].

Nephropathic cystinosis (NC) is a rare metabolic disease caused by mutations in the *CTNS* gene that encodes for cystinosin, the cystine carrier in lysosomes [108]. NC is characterized by an impaired transport of cystine out of lysosomes [109], and is the most frequent cause of Fanconi syndrome (FS) in young children. Renal FS is characterized by impaired reabsorption processes in proximal tubular cells, with subsequent loss of electrolytes, glucose, bicarbonate, phosphate, amino acids, and low molecular-weight proteins [110], leading to susceptibility towards apoptosis. In primary proximal tubular cells of the kidney from a *Ctns*-knockout mouse model [111] and in two independently human *CTNS*^−/−^ conditionally immortalized proximal tubular epithelial cells carrying pathological mutations [112], mitochondrial defects have been observed in terms of reduced complex I [111,112] and V [112] activities, reduced mitochondrial membrane potential [111,112], and increased mitochondrial fragmentation [112]. Reduction of complex I and V activities has been associated with lower expression of some of their subunits [112]. An increase of cellular levels of PHB1 protein has been found in primary proximal tubular cells of the kidney, derived from a *Ctns*-knockout mouse model, which were more susceptible towards apoptosis and presenting mitochondrial defects [111]. Since the cystinosis is a lysosomal storage disease associated with mitochondrial damage, the increased expression of PHB1, in this case, might be due to the UPR^mt^.

### 8.4. Prohibitins in Cardiac Diseases

Cardiac disease is a leading cause of morbidity and mortality worldwide. Evidences suggest a contribution of altered mitochondrial fission and apoptosis in the pathogenesis of cardiac diseases, [113] and PHBs, by modulating mitochondrial dynamics, could contribute to the pathogenesis of cardiac diseases.

Over-expression of PHB1 has been found to protect cardiomyocytes from hypoxia-induced cell death by inhibiting cytochrome *c* release and decreasing levels of Bcl2 protein [71]. In addition, over-expression of PHB1 in cardiomyocytes has been shown to protect cells from oxidative stress-induced mitochondrial apoptosis and preserve the cytochrome *c* release and mitochondrial membrane permeability [44]. Furthermore, in spontaneous hypertensive rats, in left ventricles with left ventricular hypertrophy it was found that mitochondrial dysfunction was associated with lower mRNA and mitochondrial PHB protein levels [41]. In male Sprague Dawley rats, reduced PHB protein levels have been found in isoproterenol-induced cardiac hypertrophy [114].

The decreased expression of PHBs in cardiac diseases has been studied at the molecular level, and has been associated overall with changes in the expression of non-coding RNAs. In isolated cardiomyocytes from male mice, a report showed that anoxia treatment induces apoptosis associated with mitochondrial fission and down-regulation of PHB2 in mitochondria, and the enforced expression of PHB2 ameliorates mitochondrial fission, preventing apoptosis [115]. The same results were obtained in mice using the ischemia/reperfusion (I/R) model. I/R also causes a reduction in mitochondrial PHB2 levels, and the upregulation of PHB2 ameliorates the response to I/R by reducing mitochondrial fission, apoptosis, and infarct sizes. The well-studied molecular mechanism shows that, after anoxia treatment, a reduction of a long non-coding RNA (lncRNA), named cardiac apoptosis-related lncRNA (CARL), occurs. Under normal conditions, CARL inhibits miR-539 expression. After anoxia treatment, the reduction of CARL favors the expression of miR-539 that, in turn, reduces the PHB2 expression level [115].

Other works report the role of miRNA in modulating the expression of PHBs. In response tohydrogen peroxide treatment of cardiomyocytes, mitochondrial fission and apoptosis were observed, which were associated with the decrease in PHB1 protein levels and an increase in miR361 expression. Studies in cardiac-specific PHB1 transgenic mice and miRNA361 transgenic mice suggest that the over-expression of miR-361 in response to myocardial infarction surgery reduces the translation of PHB1, favoring mitochondrial fission and myocardial infarction sizes [64].

In cardiomyocyte culture cells, isolated from neonatal rats, treatment with hydrogen peroxide or doxorubicin caused apoptosis. The induced oxidative stress resulted in up-regulation of miRNA-23a that, in turn, increased the transcriptional activity of p53 that, in turn, promoted the expression of another miRNA, miRNA-128. miRNA-128 suppressed the PHB1 expression, promoting apoptosis [78]. In rat cardiac hypertrophy induced by transverse aortic constriction, a differentially expressed long non-coding RNA (lncRNA) has been found. By co-expression network analysis, a correlation between lncRNA BCO88254 and PHB2 has been found, even if the relationship is unclear [116].

### 8.5. Prohibitins and Cancer

The important role of PHBs in mitochondrial apoptosis is underlined by the fact that in different types of cancer cells, with higher resistances to apoptosis, an increase of PHB levels has been found and, similarly, reduced PHB levels give cancer cells a higher sensitivity towards apoptosis [25,117]. Moreover, PHBs play a role in cancer cell propagation and survival [118], and down-regulation of PHB expression drastically reduces the rate of cell division. There are many studies reporting the association of PHBs with cancer. PHB protein and mRNA expressions have been found increased in gastric cancer tissues [119], esophageal squamous cell carcinoma [120], colorectal carcinoma tissues [121], human prostate cancer cells [122], high-grade breast cancer [123], human bladder cancer [124], papillary thyroid carcinomas [125], and hematologic tumor cells [126], and are risk factors for liver cancer [127,128]. We anticipate that, in human ovarian cancer tissue, compared to normal tissue, the increased level of PHB2 correlates well with the increased level of OPA1, mitochondrial fusion, and biogenesis, and decreased diameters of cristae junctions, suggesting resistance towards apoptosis [129].

Furthermore, several anticancer drugs have been found to bind to PHBs. About 30% of human carcinomas shows mutations of RAS GTPase oncogenes, leading to activation of CRAF. It has been found that augmented expression of PHB1 contributes to the increase of CRAF activity associated with enhanced migration and metastases in cervical carcinoma cells [130]. Rocaglamides and flavaglines are potent natural anticancer products that sensitize resistant cancer cells to apoptosis by targeting PHB proteins. This interaction blocks the binding of PHB to CRAF and thereby inhibits CRAF activation [131,132].

## 9. Conclusions

The above described functions underline the critical roles played by PHB proteins in mitochondrial biology, especially in mitochondrial dynamics, structure, and apoptosis. This is also highlighted by the potential roles of PHBs in different pathologies and the possibility for PHBs to be considered as molecular biomarkers and/or therapeutic targets [26,133]. Furthermore, it is clear that cancer diseases, with high cellular resistance to apoptosis, are characterized by an increased expression of PHBs, while in other diseases, such as PD, with more susceptibility towards apoptosis, a defect of PHBs is evident. However, at the same time, due to their multifunctional roles in the cell, manipulating PHB expression is likely to have unpredictable consequences, which would be difficult to predict and control. Indeed, different phenotypes have been observed upon PHB depletion depending on the metabolic status of the cells. It should also be noted that in addition to the depletion of PHBs inducing mitochondrial dysfunction and apoptosis, an increase of PHBs, which is protective against apoptosis, could be also due to mitochondrial dysfunction and thus to UPR^mt^-dependent mechanisms. Further investigations are needed to define the signaling pathways and post-translational modifications that induce the expression or re-localization of PHB proteins in the cellular compartments in different metabolic status and stress conditions.
